# The impact of an existing Anesthesia Control Tower (ACT) trial to improve intraoperative care on infectious outcomes

**DOI:** 10.1017/ash.2025.400

**Published:** 2025-09-24

**Authors:** Katelin Nickel, Jonas Marschall, Thomas Kannampalil, Thaddeus Budelier, Alexander Kronzer, Hilary Babcock

**Affiliations:** 1 University of Arizona College of Medicine – Phoenix; 2 Washington University School of Medicine

## Abstract

**Background:** Adverse perioperative outcomes remain a major public health challenge despite being largely preventable. Surgical site infection (SSI) is a common preventable healthcare-associated infection (HAI) following surgery. The Barnes-Jewish Hospital anesthesiology department developed an innovative telemedicine model, the Anesthesia Control Tower (ACT), to improve intraoperative care delivery and address predisposing factors for adverse outcomes. The ACT is staffed by anesthesiologists and certified nurse anesthetists who use a customized, electronic monitoring system, bolstered with machine-learning forecasting, to provide real-time clinical decision support to anesthesia providers in the operating room (OR). In the ACT randomized control trial, alerts are generated during surgical procedures and ORs are randomized to receive ACT input based on alerts or not. We were interested in determining the effect of the ACT on infectious outcomes. **Methods:** We used the existing ACT study design, a randomized control trial, to determine the impact of the ACT monitoring system above on SSI, CLABSI and CAUTI. HAI surveillance was performed by IP specialists using CDC NHSN definitions for SSI, CLABSI and CAUTI. We included CABG, colon, abdominal hysterectomy, hip, and knee arthroplasty procedures performed during ACT hours of operation from July 1, 2018 to January 31, 2023 and compared outcomes among patients by randomization to receive ACT. Here, we report on the intention-to-treat analysis based on randomization status. **Results:** The final cohort included 8,993 procedure dates including 862 CABG procedures, 2,654 colon surgeries, 2,732 abdominal hysterectomies, 2,105 hip arthroplasties, and 833 knee arthroplasties. Baseline characteristics (e.g. age, comorbidities, wound class) were balanced by randomization status. Characteristics captured during the procedure (e.g., temperature, oxygen) were also similar by randomization status. The infectious outcomes revealed that there was no difference in likelihood of SSI (4.0% vs 4.0%), 60-day CLABSI (0.2% vs 0.3%), or 60-day CAUTI (0.0% vs 0.1%) whether the procedure was randomized to receive ACT input or not. Thirty-day mortality (2.0% vs 1.9%) and readmission (16.6% vs 15.9%) did not differ by randomization. **Conclusions:** In this intention-to-treat analysis of the impact of a novel anesthesiology monitoring system on HAI outcomes, we did not find a difference in the incidence of SSI, CLABSI or CAUTI. Next, we will analyze the study data in a per-protocol fashion. This RCT was conducted in a resource-rich environment with robustly implemented best practice where a second layer of anesthesiology supervision may confer little benefit. The concept of an ACT may still be helpful in resource-limited settings.

